# Comparative genomic analysis of 142 bacteriophages infecting *Salmonella enterica* subsp. *enterica*

**DOI:** 10.1186/s12864-020-6765-z

**Published:** 2020-05-26

**Authors:** Ruimin Gao, Sohail Naushad, Sylvain Moineau, Roger Levesque, Lawrence Goodridge, Dele Ogunremi

**Affiliations:** 1 Ottawa Laboratory Fallowfield, Canadian Food Inspection Agency, Ottawa, Ontario Canada; 2 Department of Food Science and Agricultural Chemistry, McGill University, Ste Anne de Bellevue, QC Canada; 3 Félix d’Hérelle Reference Center for Bacterial Viruses, Faculté de médecine dentaire, Université Laval, Québec City, QC G1V 0A6 Canada; 4 Groupe de recherche en écologie buccale, Faculté de médecine dentaire, Université Laval, Québec City, QC G1V 0A6 Canada; 5 Département de biochimie, de microbiologie, et de bio-informatique, Faculté des sciences et de génie, Université Laval, Québec City, QC G1V 0A6 Canada; 6 Institut de Biologie Intégrative et des Systèmes, Université Laval, Québec City, QC G1V 0A6 Canada; 7 Present Address:Department of Food Science, University of Guelph, Guelph, Ontario Canada

**Keywords:** Comparative genomics, Bacteriophage, Nucleotide identity, *Salmonella enterica*, Phamerator, Prophage sequence typing, Phage clusters

## Abstract

**Background:**

Bacteriophages are bacterial parasites and are considered the most abundant and diverse biological entities on the planet. Previously we identified 154 prophages from 151 serovars of *Salmonella enterica* subsp. *enterica*. A detailed analysis of *Salmonella* prophage genomics is required given the influence of phages on their bacterial hosts and should provide a broader understanding of *Salmonella* biology and virulence and contribute to the practical applications of phages as vectors and antibacterial agents.

**Results:**

Here we provide a comparative analysis of the full genome sequences of 142 prophages of *Salmonella enterica* subsp. *enterica* which is the full complement of the prophages that could be retrieved from public databases. We discovered extensive variation in genome sizes (ranging from 6.4 to 358.7 kb) and guanine plus cytosine (GC) content (ranging from 35.5 to 65.4%) and observed a linear correlation between the genome size and the number of open reading frames (ORFs). We used three approaches to compare the phage genomes. The NUCmer/MUMmer genome alignment tool was used to evaluate linkages and correlations based on nucleotide identity between genomes. Multiple sequence alignment was performed to calculate genome average nucleotide identity using the Kalgin program. Finally, genome synteny was explored using dot plot analysis. We found that 90 phage genome sequences grouped into 17 distinct clusters while the remaining 52 genomes showed no close relationships with the other phage genomes and are identified as singletons. We generated genome maps using nucleotide and amino acid sequences which allowed protein-coding genes to be sorted into phamilies (phams) using the Phamerator software. Out of 5796 total assigned phamilies, one phamily was observed to be dominant and was found in 49 prophages, or 34.5% of the 142 phages in our collection. A majority of the phamilies, 4330 out of 5796 (74.7%), occurred in just one prophage underscoring the high degree of diversity among *Salmonella* bacteriophages.

**Conclusions:**

Based on nucleotide and amino acid sequences, a high diversity was found among *Salmonella* bacteriophages which validate the use of prophage sequence analysis as a highly discriminatory subtyping tool for *Salmonella.* Thorough understanding of the conservation and variation of prophage genomic characteristics will facilitate their rational design and use as tools for bacterial strain construction, vector development and as anti-bacterial agents.

## Background

The Gram-negative bacterial genus *Salmonella* belongs to the family Enterobacteriaceae, order Enterobacteriales, class Gammaproteobacteria and phylum Proteobacteria. *Salmonella* cells have a length of 2 to 5 μm and a diameter ranging from 0.7 to 1.5 μm, as well as being predominantly motile due to peritrichous flagella [1]. The genus consists of two species, namely *Salmonella enterica* and *S. bongori*. The former can be further divided into six subspecies which corresponds to known serotypes (depicted with Roman numerals): *enterica* (I), *salamae* (II), *arizonae* (IIIa), *diarizonae* (IIIb), *houtenae* (IV) and *indica* (VI) [2]. The serotype V is now considered a separate species and designated *S. bongori*. Based on the presence of somatic O (lipopolysaccharide) and flagellar H antigens (Kauffman-White classification), the above six *S. enterica* subspecies are divided into over 2600 serovars [3] but fewer than 100 serovars have been associated with human illnesses [4]. *Salmonella enterica* subpecies *enterica* is typically categorized into typhoidal and non-typhoidal *Salmonella* as a result of symptoms presenting in infected humans. Non-typhoidal *Salmonella*, which is made up of a large number of the serovars, can be transmitted from animals to humans and between humans, often via vehicles such as foods, and they usually invade only the gastrointestinal tract leading to symptoms that resolve even in the absence of antibacterial therapy [5]. In contrast, typhoidal *Salmonella* serovars such as Typhi, Paratyphi A and Paratyphic C, are transferred from human to human and can cause severe infections requiring antibiotic treatment [6]. Wide spread resistance against antibiotics has prompted a renewed surge of interest in bacteriophages which are viruses capable of infecting and sometimes killing bacteria, as safe and effective therapy alternatives [7].

Bacteriophages, sometimes simply referred to as phages, are considered the most abundant biological entities on the planet [8]. These bacterial viruses can undergo two life cycles: lysis or lysogeny. A bacteriophage capable of only lytic growth is described as virulent. In contrast, temperate bacteriophage refers to the ability of some phages to display a lysogenic cycle and instead of killing the host bacterium becomes integrated into the chromosome. A bacterium that contains a set of phage genes representing an intact prophage is called a lysogen, while the integrated viral DNA is called a prophage. Most temperate phages form lysogens by integration at a unique attachment site in the host chromosome [9, 10]. The integration process has been described as a biological arms race between the infecting virus and the host bacterium [11]. There is an array of host defense mechanisms that are stacked against the virus which in turn increasingly acquires and displays a counter-offensive to thwart and evade the anti-viral mechanisms resulting in integration into the host genome [11–13].

Tailed phages which belong to the Order Caudovirales are the most abundant group of viruses infecting bacteria and are also the most prevalent in the human gut. They are easily recognized under an electron microscope by their polyhedral capsids and tubular tails [14]. The order Caudovirales is made up of five families, namely: (1) *Myoviridae* (contractile tails, long and relatively thick), (2) *Siphoviridae* (long noncontractile tails), (3) *Podoviridae* (short noncontractile tails) [14, 4) *Ackermannviridae* (contractile tails) and (5) *Herelleviridae* - spouna-like (contractile tails, long and relatively thick) [15]. Bacteriophages were first described by Frederick Twort in 1915 and Felix d’Herelle in 1917 [16], and studies into their relationship with *Salmonella enterica* serovar Typhimurium led to the description of “symbiotic bacteriophages” by Boyd [17]. We recently analyzed the bacteriophages present in 1760 genomes of *Salmonella* strains present in a research database (https://salfos.ibis.ulaval.ca/) and apart from three strains devoid of any prophage, the genomes had 1–15 prophages with an average of 5 prophages per isolate [18]. Previous analyses of *Salmonella* phages have led to their classification into five groups (P27-like, P2-like, lambdoid, P22-like, and T7-like) and three outliers (ε15, KS7, and Felix O1) [10]. Apart from the primary role of phage gene products to ensure that these viruses can infect bacteria, survive and reproduce in their hosts, phage genes have been shown to code for virulence factors, toxin, and antimicrobial resistance genes. The presence of these genes appears to contribute in a substantial manner to the evolution of the bacterial host [18–20]. Studies of prophage biology have practical significance in choice of phages as antibacterial agents, in bacterial strain construction and typing for epidemiological purposes [21, 22].

The advent of whole genome sequencing has greatly facilitated the detection and characterization of phages and prophages in bacterial hosts and the ability to evaluate their impacts on the host. Evolutionary analysis of phage genes open reading frames (ORF) families based on sequence analysis of a large number of phage genomes in the GenBank (about 13,703 phage genomes were present as of June 2019) (http://millardlab.org/bioinformatics/bacteriophage-genomes/phage-genomes-june-2019/) has provided insights into the impact on the evolution of both the virus and host [23]. Whole-genome comparative analysis has been successfully applied to study phages present or infecting several bacterial genera including *Mycobacteria* [24], *Staphylococcus* [25], *Bacillus* [26], *Gordonia* [27], *Pseudomonas* [23] and as well as the *Enterobacteriaceae* family [28]. Phage genomes are commonly grouped into clusters, but outlier phages lacking strong nucleotide identity relationships with other clustered genome are often designed as ‘singletons’ [27]. To classify phage genomes into clusters and subclusters, there are several commonly used tools/approaches. The dot plot program Genome Pair Rapid Dotter (Gepard) [29] can reveal very substantial synteny among genomes. Typically, the dot plot can recognize similarities spanning more than half of the genome lengths [24]. The average nucleotide identity (ANI) are determined using tools such as Kalign [30] and MUMmer [31] using genomes alignment and comparison. Genome map and gene content analyses can be performed using Phamerator, which assorts protein-coding genes into Phamilies (Phams) and generate a database of gene relationships [32, 33].

Using PHASTER (PHAge Search Tool Enhanced Release) [34, 35], we previously demonstrated the presence of 154 different prophages in 1760 *S. enterica* genomes which covered 151 *Salmonella serovars* [18]. We also previously showed that some prophage sequences were conserved among strains belonging to the same serovars and that the prophage repertories provided an additional marker for differentiating *S. enterica* subtypes during foodborne outbreaks [18]. Here, a more detailed characterization of these *Salmonella* phage genomes was carried out to generate knowledge on their biological variation and evolution and thereby provide insights into the role of phages in *S. enterica* taxonomy, diversity and biology.

## Results

### 142 *Salmonella* phage genome sequences and patterns of variation

**Fig. 1:**
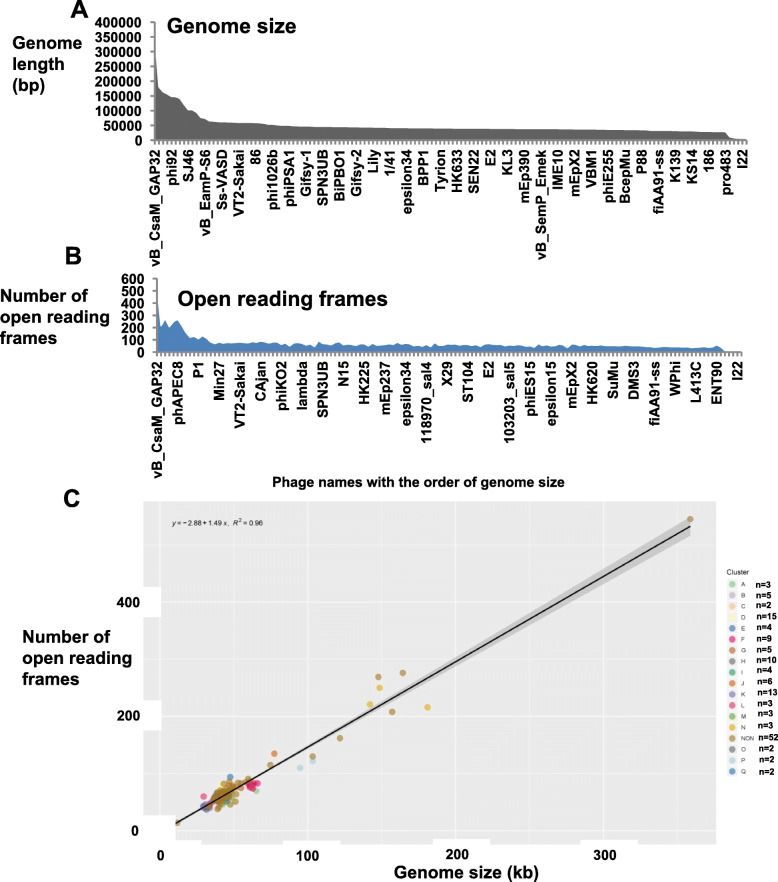
Genome characteristics of 142 *Salmonella* prophages. **a** Plot of genome sizes **b** Plot of the number of Open Reading Frames (ORFs). X axis shows names of each of the 142 prophages. Y axis represents either the genome length or number of detected ORFs in each prophage genome. **c** The correlation between the number of predicted ORFs and genome size in prophage genomes (R^2^ = 0.95, *p* < 0.001). The shading besides the line indicates 95% confident interval of the linear correlation. The genomes from different clusters were shown with a different color of dot

Complete genome sequences of *S. enterica* prophages were searched and downloaded from the NCBI database. Full genome sequences were available for 142 phages (Document S1) and their corresponding genomic information are summarized in Table S1 and include accession number, phage name, assigned cluster, host species, genome size, guanine plus cytosine (GC) content, number of ORFs and virus lineage and DNA structure, i.e., double stranded (dsDNA) or single stranded (ssDNA). The annotated information for the 142 phage genomes was summarized in Document S2. The size range of the phage genomes was from 6.4-kb to 358.7-kb, with the majority between 30-kb to 50-kb (Fig. 1a), the GC content ranged from 35.5 to 65.4% (Table 1 & S1). The virus lineages for all 142 phages were summarized in Table 1 & S1. Ninety-five percent of the phage genomes (135 out of 142) were linear ds DNA and belong to the order Caudovirales and four out of its five known families, namely: *Myoviridae*, *Siphoviridae*, *Podoviridae* and *Ackermannviridae* based on virus lineages retrieved from Virus-Host DB. There is a total of 27 genera represented in this collection of 142 prophages (Table 1). Four of the remaining seven phages (5%) were single stranded DNA (NC_001954.1, NC_006294.1, NC_001332.1 and NC_025824.1), while three have not yet been classified (NC_010393.1, NC_010392.1 and NC_010391.1).

**Table 1:** The characteristics of 142 prophages present in *Salmonella enterica*

Characters	Range or Number
Genome size (bp)	From 6408 to 358,663
GC (%)	From 35.5 to 65.4
Open Reading Frame	From 9 to 545
Clusters	15
Prophage lineage_Family	5
Prophage lineage_Genus	27
Original host lineage_Family	15
Original host lineage_Genus	24

### Open reading frame characterization of phage genomes

The availability of the 142 phage sequences in the NCBI database facilitated comparative genomic analysis. However, 32 out of 142 phages downloaded from the GenBank contained invalid start or stop codons for some ORFs, which were detected during our construction of the *Salmonella* prophage database (SpDB) and analysis with the Phamerator software (see under Materials and Methods). To ensure congruence between the annotations shown in the GenBank and ORFs displayed by the Pharmerator, it became necessary to ensure that proper start and stop codons were present in the sequences. The detailed error messages (including number of errors and their locations in the original sequences) are shown in Table S1, and the revised sequences and NCBI files are now included in Document S2. The distribution of the genome sizes mirrored the number of ORFs, with the genome size (grey) matching the number of ORFs (blue) as displayed in Fig. 1a and b. For instance, the 4 genomes with the smallest size (6408, 6744, 7107 and 8454 bp) had the least ORFs (10, 9, 12, and 10, respectively). Similarly, the 10 largest genomes encoded the highest number of ORFs, typically over 120 ORFs (Table S1). There was a statistically significant, strong linear correlation between the genome sizes and number of ORFs (R^2^ = 0.95, *p* < 0.001, Fig. 1c).

### *Salmonella* phages occur in other bacteria

**Fig. 2:**
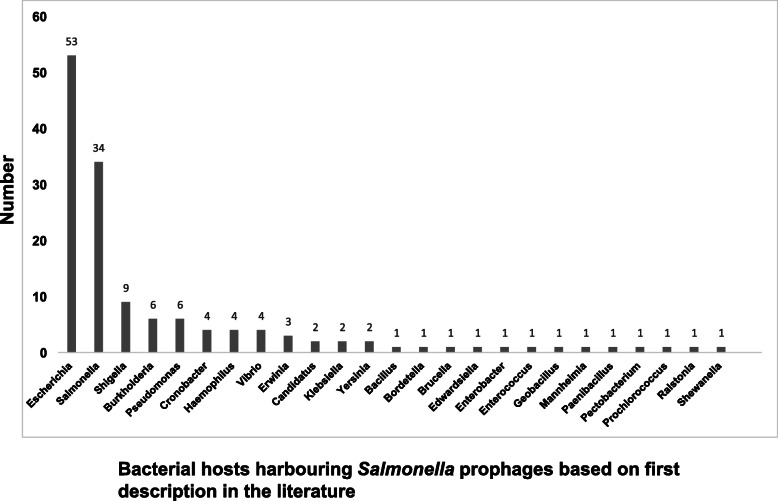
Bacterial hosts of 142 *Salmonella* prophages. The X axis represents the number of prophages while the Y axis represents the frequency of occurrence in the bacterial host as identified in Virus-Host DB (https://www.genome.jp/virushostdb/)

Although the 142 prophages were identified in *Salmonella enterica* strains present in the Salfos database [17], many prophages matched sequences of viral origin associated with bacterial hosts other than *Salmonella*. This designation of a non-*Salmonella* host was presumably a consequence of which host the prophage was associated with at the time of initial documentation or publication. The original known host lineage for each phage was used to evaluate the occurrence of these phages in other bacteria. As shown in Table S1 and illustrated in Fig. 2, fifty-three out of the 142 *Salmonella* phages (37.3%) were apparently first recovered from the genus *Escherichia*, followed by 34 phages (23.9%) first described for a *Salmonella* host. The others, including *Shigella, Burkholderia,* and *Pseudomonas,* showed relatively lower frequencies of 9, 6, and 6 phages, respectively (Fig. 2). Although the cellular host for the phage P4 is named as *Escherichia*, it is indeed a satellite virus for another phage called *Escherichia* virus P2, the latter serving as a helper to provide late gene functions for phage P4 lytic growth cycle, but not for its early functions especially DNA synthesis and lysogenization [36, 37]. The host of each prophage was detected at a 97% agreement with the metadata on the bacterial host documented in the Virus-Host Database (Table S1).

### Similarities among the 142 phage genomes based on nucleotide identity

**Fig. 3:**
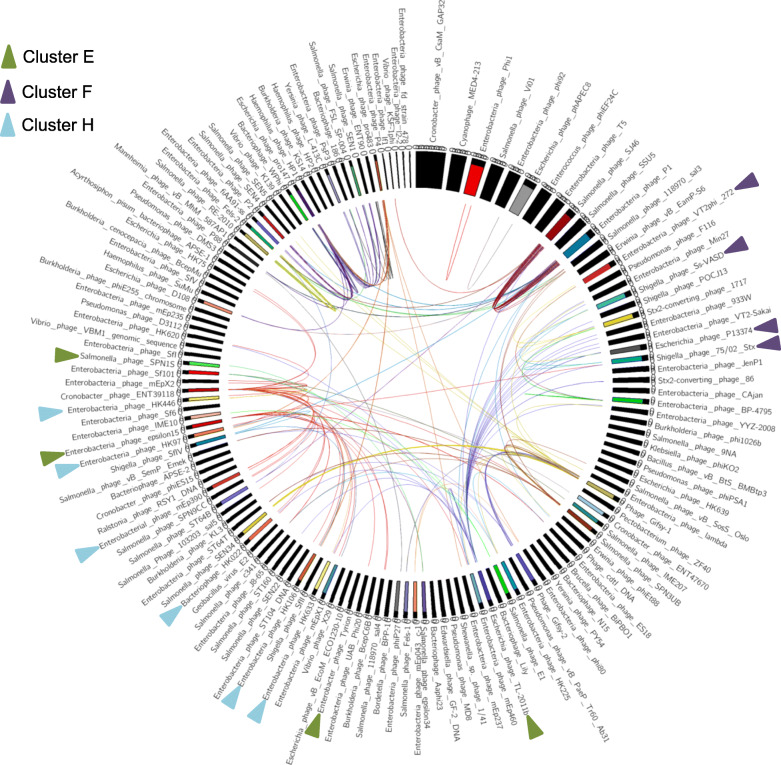
Similarities among 142 *Salmonella* prophages based on nucleotide identity and displayed using Circos. Nucleotide identities between prophages were calculated and coordinates were generated using NUCmer/MUMmer and displayed as Circos. Names of prophages are shown on the outer layer and arranged according to genome sizes. Prophages are highlighted in color block if more than one link (using the same color line as prophage block) existed with any of the other prophages. In contrast, prophages were shown in black block if no nucleotide similary was detected with the other genomes

Given that nucleotide identity and genome alignment are key tools for comparative genomic analysis and cluster assignment, NUCmer/MUMmer software was initially applied to analyze these 142 prophage sequences. The pairwise nucleotide identity was calculated among all the 142 genomes and those fragments with over 80% identity between two genomes were listed in Table S2. The sizes of aligned phage genome fragments varied, ranging from 103 bp to 14,505 bp. Out of the 142 genomes investigated, 133 shared at least one fragment with another prophage. We found two phage genomes namely, *Salmonella*_phage_SJ46 (103 kb) and *Enterobacteria*_phage_P1 (95 kb), to share an exceptionally large number of fragments with other *Salmonella* prophages as shown in Fig. 3. In a striking contrast, *Salmonella*/*Cronobacter* prophage vB_CsaM_GAP32 and *Salmonella*/cyanophage MED4–213, which have the two biggest genomes (181- and 359-kb) did not share any fragment with another phage genome.

### Clustering of phage genomes

**Fig. 4:**
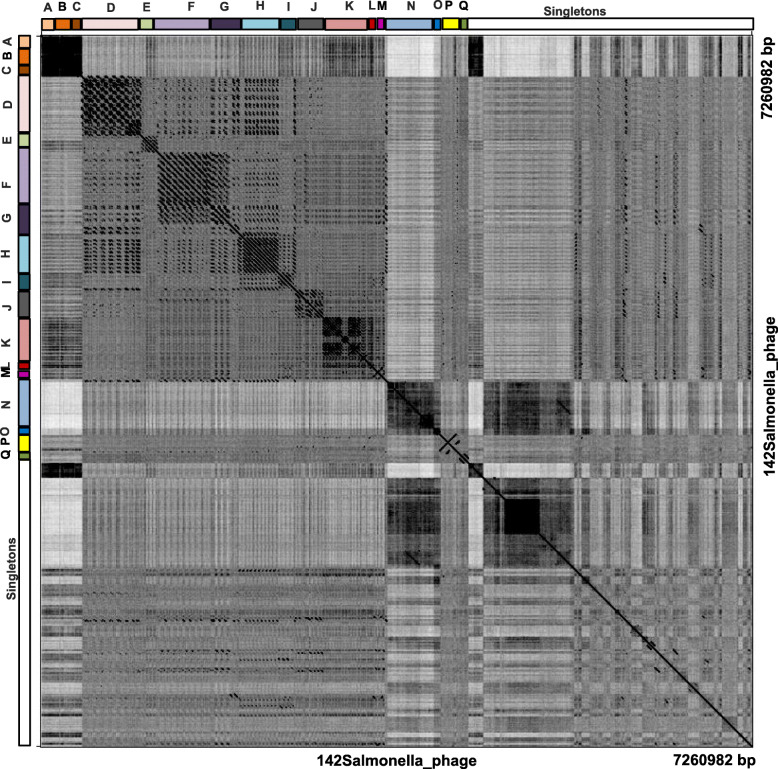
Whole-genome dot plot comparison of prophage nucleotides sequences of *Salmonella*. Prophage genomes (*n* = 142 phage) were concatenated into a single sequence with a total length of 7,260,982 bp, which plots against itself with a sliding window of 10 bp and visualized by Genome Pair Rapid Dotter (Gepard) 1.40 version. A total of 90 prophage genomes were assigned to 17 groups **a** - **q**, and the remaining 52 prophage genomes plotted as singletons

Conserved DNA fragments among groups of prophage sequences (Fig. 3), were combined with the results of ANI and whole genome dot plot analysis, to assign the prophage genomes to clusters. To this end, a phylogenetic tree from the genome nucleotide identity matrix generated with the Kalign algorithm (Fig. S1). Furthermore, all 142 genomes were concatenated into a single nucleotide sequence and duplicated to form two axes for the purpose of generating a dot plot matrix (Fig. 4). We were able to assign 90 phage genomes into 17 clusters, named A to Q as follows: Cluster A (*n* = 3), Cluster B (*n* = 5), Cluster C (*n* = 2), Cluster D (*n* = 15), Cluster E (*n* = 4), Cluster F (*n* = 9), Cluster G (n = 5), Cluster H (*n* = 10), Cluster I (n = 4), Cluster J (*n* = 6), Cluster K (*n* = 12), Cluster L (n = 3), Cluster M (n = 3), Cluster N (n = 3), Cluster O (n = 2), Cluster P (n = 2) and Cluster Q (n = 2). The remaining 52 phage genomes could not be assigned to any cluster and remained as singletons. We observed both qualitative and quantitative differences in the structure of the clusters based on the intensity of the dot plots (Fig. 4) and pairwise nucleotide similarity between members of each cluster (Table S3, Cluster A-Q). Clusters E, F, H, I and J had relatively high intracluster nucleotide similarities and moderate genome sizes (37–77 kb). All four members of Cluster E belonged to the same genus, Epsilon15 virus under the family of *Podoviridae* according to the International Committee on Taxonomy of Viruses (ICTV) classification. Details of cluster assignment for all prophages are shown in Table S1. We observed uniformity among the genome sizes and number of ORFs of members of the same cluster (Fig. 1c) which underscores the nucleotide identity among related genomes as also shown in Fig. 3.

### Genome maps of multiple phages that incorporate and display nucleotide and amino acid sequence relationships

**Fig. 5:**
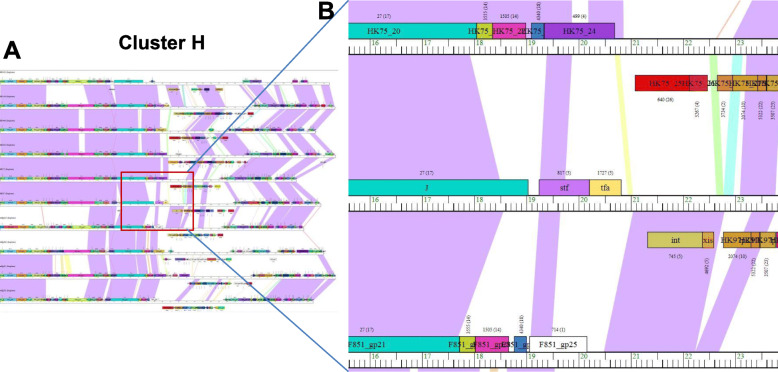
Genomic maps of *Salmonella* prophages belonging to Cluster H using the Phamerator software. **a** 11 prophage genomes (mEp390, mEpX2, HK75, mEpX1, HK633, HK446, mEp235, ENT39118, HK022, HK97 and HK106) was present in Cluster J. **b** Close-up view of partial of cluster J map. Blocks represent predicted ORFs, genes are color-coded according to their pham assignment. Gene names are shown within each gene box and the pham number and number of pham members are shown in parentheses above each gene. Shading between genomes indicates regions of pair-wise nucleotide similarity and was coded in color spectrum so that color indicates nucleotide similarity with violet being the most similar and red being the least similar. No shading suggests there is no similarity

Using a ClustalW threshold of 35% amino acid identity and a BLASTP score of 1e-50, the predicted ORFs and translated nucleotide sequences were assigned to groups of closely related sequences using the Phamerator software (Document S3 and Fig. 5). A total of 5796 Phamilies was assigned by Phamerator (Table S4). The most common Phamily was present in 49 prophages but there were 4330 Phamilies found in only one prophage. The relatively conserved Phamily numbers were summarized in the 17 assigned clusters in Table 2 & S5. To establish cluster-specific markers, we retrieved the conserved phamilies from each analyzed clusters and found that a total of 181 representative protein groups were present in all 17 clusters and 159 of them (excluding the 22 bold highlighted proteins in Table S5) were specifically present in one cluster. For example, Cluster A uniquely contained seven Phamilies. In contrast, Cluster H contained 10 Phamilies but not all were unique because two of these Phamilies were also present in Cluster I. In the same vein, Cluster K contained 15 Phamilies, seven of which were shared with Cluster L. Thus, we demonstrated the presence of unique proteins and/or unique combination of proteins that define each prophage cluster, notwithstanding the fact that some individuals’ proteins may be shared among some clusters. A representative genomic map of phages in Cluster H is shown in Fig. 5. Considerable genome length was observed to be conserved between members of the same cluster inferring synteny (violet shading blocks), with the same phamily ORF (same colour, Fig. 5). Often syntenic regions are interspersed with dissimilar and variable sequences (white blocks or breaks).

**Table 2:** The distribution of conserved Phamily members (*n* = 181) among clusters of *Salmonella* bacteriophages

Cluster	No. of Pham	Cluster	No. of Pham	Cluster	No. of Pham
A	3	G	5	M	3
B	5	H	10	N	3
C	2	I	4	O	2
D	15	J	6	P	2
E	4	K	12	Q	2
F	9	L	3		

## Discussion

We have carried out a comparative genomic analysis for the purpose of characterizing the prophages of *Salmonella enterica*. Both dsDNA and ssDNA viruses were represented in our collection of 142 phage genomes. The four ssDNA phages present in our collection belonged to the family *Inoviridae.* In contrast, the dsDNA phages were spread over four of the five known families of the order Caudovirales, i.e., *Myoviridae, Podoviridae, Siphoviridae* and the rare *Ackermannviridae*. Within these four families, a total of 27 different phage genera were represented (Table 1). Earlier studies using core genes analysis indicated that *Salmonella* phages could be classified into five groups, namely: P27-like, P2-like, lambdoid, P22-like, and T7-like [9, 10], and all of which were present in our prophage collection. From our classification, we have identified two new members of Cluster D namely, ST64T and ST104 which are related to the previously described P22-like group. We have described an additional 13 members in this group (Table S1). Similarly, we detected the P2-like PSP3 phages and were able to cluster them with an additional 12 double stranded phage viruses to make up Cluster K. In addition, three lambdoid phages, namely Gifsy 1, Gifsy 2 and lambda were assigned to lambdoid phage group Cluster M (Table S1). This work has extended published observations by identifying additional members of previously described, albeit small groupings, and has achieved a more discriminative and extensive characterization of *Salmonella* prophage sequences.

An earlier genomic comparison of tailed phages showed 337 fully sequenced lytic and temperate phages in the entire Enterobacteriaceae family [28], and based on this observation, a large number of diverse phages could potentially infect *Salmonella*. We observed the presence of the same phages infecting different bacteria and whether this is an outcome of the shared location or relatedness among hosts cannot be ascertained at this time. It is possible that both phylogeny, i.e., the relatedness among hosts such as belonging to the same family, or occupation of the same niche, i.e., gastrointestinal tract location may facilitate the presence of same prophages in different hosts. As examples, we observed phages X29 and KSF-1phi in *Salmonella*, which were first found in *Vibrio cholerae* according to Virus-Host DB [TAX:666; https://www.genome.jp/virushostdb/]. On the other hand, 38 other phages known to infect *Vibrio cholera* have not been reportedly found in *S. enterica* and given that the two organisms belong to different orders, this suggests that hosts phylogeny rather than co-location plays the primary role whether prophages are shared among hosts. Nevertheless, it is difficult to entirely discount the role of a shared niche since the virus will still have to find the new host before infection can take place. Furthermore, 33 phages analyzed here were observed to have originated from *Escherichia coli* strains [TAX:562] (Table S1). *Enterobacteria* phage fiAA91-ss is also able to infect at least two more hosts, namely, *Shigella sonnei* [TAX:624] and *Escherichia coli* O157:H7 [TAX:83334]. *Haemophilus* phage Aaphi23 can also infect *Aggregatibacter actinomycetemcomitans* [TAX:714] and *Haemophilus* [TAX:724]. The species A. *actinomycetemcomitans* has now been renamed *Haemophilus actinomycetemcomitans* by Potts et al. (1985) [38]. Based on our observations, studies of phage host range should not be restricted to specific species but should comprehensively involve as many different host genera as possible to capture all available information, even if the focus is a particular host species. This will help provide a broader perspective of the distribution of phages and understand how they contribute to the evolution of the host.

The occurrence of the same phage sequences in different hosts may also imply horizontal viral gene transfer among hosts belonging to different genera. Genome clustering facilitates the identification of genes that are in greatest genetic flux and are more likely to have been exchanged horizontally during a relatively recent evolutionary time. Such viral sequence exchanges may help a phage increase its fitness to invade a new host, and evade selective pressure such as anti-phage defense mechanisms [11]. Given the biological arms race between bacteria and phages, and in order to thrive in most environments, phages have evolved multiple tactics to avoid, circumvent or subvert bacterial anti-phage mechanisms [21]. Ironically, these viral sequences once established in *Salmonella* may help the host to thrive in specific ecological niches, including the gut [39].

Diverse phage genomes were identified in our *Salmonella* phage collection. As shown in Fig. 2, the highest number of matching prophages were named after the genus *Escherichia* (*n* = 53) while *Salmonella* ranked second (*n* = 34). Regarding the lineage for their original known host, three phyla (Firmicutes, Proteobacteria and Cyanobacteria), four classes (Bacilli, Betaproteobacteria, Alphaproteobacteria and Gammaproteobacteria) and 24 unique genera could be identified (Table 1). Such a wide host span provides further evidence of the diversity of *Salmonella* prophages analyzed in this study. In a study of prophages integrated in a single host species *Mycobacterium smegnatis,* a threshold of 50% nucleotide identity was used for genome cluster assignment [24]. The threshold was slightly reduced (45%) for clustering *Pseudomonas* phages because phages infecting a genus would be expected to show greater variation in genome sequences than one infecting a single species [23]. Among the 56 phage clusters reported for the Enterobacteriaceae family, the sequence similarity was substantially less between clusters [28], indicating a higher degree of variation and justifying a lower threshold of nucleotide identity for certain clusters in *Salmonella* phages, a large proportion of which may infect or have previously infected other hosts.

It should be noted that nucleotide identity is not the only parameter for assessing genome properties, because the nucleotide alignments for thousands of homologous protein are not significant based on nucleotide alignment, but are clearly homologous based on statistically significant protein structural similarity or strong sequence similarity to an intermediate sequence [40]. Thus, there may not be a linear relationship between sequence identity and function [41]. In our set of phage genomes, except for Cluster B, L and M showed a lower pairwise ANI of 41%, all the other clusters Clusters E (59%), F (75%) and J (57%) displayed high nucleotide identity (Table S3). Their assignment to each of these clusters was supported by results of analysis using the dot plot program, Kalign genome alignment and gene content analysis. For instance, the dotplot (Fig. 4) and Kalign analyses grouped members of Clusters B, G and N, even though some of their respective nucleotide identities were 40.7, 42.2, and 42.3% (Fig. 4 and Figure S1). A similar phenomenon was also observed for Cluster L made up of members belonging to the same P2 virus group showing a nucleotide identity of 41.3%. The differences in the output of the different tools should not be surprising because of their unique underlying algorithms. While Kalign focuses more on analyzing larger genomes in general, MUMmer focuses more on the similar DNA fragment identification. Despite the high degree of diversity in our prophage collection, we were still able to cluster related isolates using congruent results from at least two bioinformatics analyses.

The genome size ranges of the prophages documented for the different bacteria genera are fairly similar: *Salmonella* (6.4–358.7 kb), *Pseudomonas* (3.0–316.0 kb), *Staphylococcus* (15.6–138.7 kb), *Gordonia* (17.1–103.4 kb), *Bacillus* (14.3–497.5 kb) and *Mycobacterium* (41.9–164.6 kb). The ranges of the GC content showed less of an overlap: *Salmonella* (35.5 to 65.4%), *Pseudomonas* (37.0 to 66.0%), *Staphylococcus* (29.3 to 38.0%), *Gordonia* (47.0 to 68.8%), *Bacillus* (29.9 to 49.9%) and *Mycobacterium* (56.3 to 69.1%) [23–27]. *Salmonella* and *Pseudomonas* both belong to the Enterobacteriaceae family and their phages share very similar genome sizes and GC content. Despite the similarities between the phages of *Pseudomonas* and *Salmonella,* the former appears to display better clustering pattern (fewer singletons) based on the grouping of 100 out of 130 phages [23] compared to 90 out of 142 *Salmonella* phages with 52 singletons. However, as *Pseudomonas* bacteriophages were collected only using “*Pseudomonas*” as host for the search in the database [23], the set most likely did not represent the full complement of viruses capable of infecting *Pseudomonas* and integrating into the genome and would have excluded bacteriophages of this group but first found or described in another bacterial host. We expect that more diverse prophage patterns would be obtained for *Pseudomonas* and other bacterial hosts if a more comprehensive search of bacterial genomes is carried out with tool such as PHASTER [34].

The diversity of *Salmonella* prophage genomes was also reflected in the total number of phamilies for the ORFs in the analyzed prophage genomes: 5796. One phamily with a Pham number of 2217 was observed to be dominant and was present in 49 prophages (34.5% of 142 phages) whereas 4330 phamilies were each present in a single prophage, which makes it challenging to select conserved genes for all the 142 prophage genomes. Clustering of the viral genome was useful in establishing relatedness of *Salmonella* bacteriophages. In each assigned cluster, some conserved Pham numbers (containing different ORFs) are present. For example, Pham 180 (portal protein), Pham 2012 (recombination protein) and Pham 2217 (endopeptidase) are commonly present in Cluster D; Pham 321 (phage head-tail connector protein), Pham 415 (terminase large subunit) and Pham 1522 (terminase small unit) in Cluster E; Pham 1995 (lysozyme), Pham 2370 (terminator) and Pham 1332 (attachment invasion locus protein precursor) in Cluster F; Pham 27 (phage tail protein), Pham 519 (phage portal protein), and Pham 1717 (assembly protein) in Cluster H; Pham 528 (major capsid protein), Pham 297 (terminase large subunit) and Pham 666 (tail protein) in Cluster J; Pham 963 (base plate assembly protein) in Cluster K (Document S3). Specifically, some proteins are unique to one cluster, for example, four members of Pham 4878 (a hypothetical protein), Pham 1893 (a hypothetical protein) Pham 2968 (a hypothetical protein) Pham 2849 (a hypothetical protein) in the Cluster E. These may be good markers for characterizing prophage members of the different clusters (Document S3, Table S5).

The observations reported in this study are quite relevant for the application of bacteriophages as antibacterial agents and in cloning vector construction. Our list of *Salmonella* bacteriophages can be used for screening a novel, candidate bacteriophage identified as a potential anti-bacterial agent for *Salmonella* or any host described in this study. The implication is that because the bacteriophages present in our collection induce lysogeny, the bacterial host will be immune to infection or lysis by the same bacteriophage; a bacteriophage on our list will likely not be an effective antibacterial agent for the hosts identified in this study. Thus, a distinct bacteriophage may be a better anti-bacterial candidate than one on our list. Similarly, the *Salmonella* prophage database in the Pharmerator can be used to evaluate a candidate antibacterial agent even if it is distinct from members on our list. Because bacteriophages are prone to recombination leading to a mosaic profile, the protein components can be used to assess relatedness with the goal of choosing a candidate antibacterial agent that is phylogenetically distant from any of the isolates in our collection to increase the chance of success. In the same vein, knowledge from our collection can be used in strategies to design phage vectors. For example, λ cloning vectors require a lytic cycle and their ability to package large foreign DNA fragments have relied on the removal of lysogenic genes from the vectors. Thus, the removal of lysogenic fragments in a temperate phage can probably deviate the life cycle into a lytic path making them more relevant for vector construction especially if the bacteriophage has signature genetic markers that can be exploited for selection or vector purification, e.g., antibacterial resistance genes or a target for a widely used ligand.

## Conclusions

The comparative genomic analysis of 142 *Salmonella enterica* subsp. *enterica* prophages revealed a high diversity in genomic characteristics, compared to that in other bacteria species such as *Pseudomonas*, *Staphylococcus*, *Gordonia*, *Bacillus* and *Mycobacterium*. The combination of nucleotide identity, dot plot, genome map comparison and gene content analysis, revealed the presence of 17 main clusters of *Salmonella* phages and many singletons. In order to have a fuller picture of *Salmonella* phages, a similar comparative phage genomic analysis needs to be performed on *Salmonella* virulent/lytic phages. The high diversity among prophages may well be a mechanism developed to generate new molecules and decoys to thwart the potent, anti-viral defence mechanism of the bacterial host. We hypothesize that in place of the resources needed to lyse a host cell, temperate prophages may instead have developed a rather sophisticated capacity to acquire and display diversity and thereby present a degree of invincibility against the host arsenal so that they can survive long enough to integrate into the host genome. Thus, we predict that prophages will show more diversity than their virulent phage counterparts. Areas of conservation and variations among the investigated prophage genomes provides further evidence showing why prophage typing is a discriminative method for *Salmonella* typing. A fuller understanding of the genomic architecture of *Salmonella* bacteriophages should furnish practical information relevant for bacterial strain construction, vector development, and the selection of appropriate phages to be tested for bio-control strategies.

## Materials and methods

### Phage genome sequences

We previously identified 154 different prophages among 1760 *S. enterica* genomes derived from 151 serovars using PHASTER [18]. We downloaded 142 of these 154 phage genomes from NCBI Batch Entrez (https://www.ncbi.nlm.nih.gov/sites/batchentrez) but were unable to locate the full length of the remaining 12 genomes. Genome annotations were downloaded from NCBI and validated using gene calling programs GeneMarkS and Glimmer [42–44], and BLASTN when necessary. The virus lineages information for the 142 prophage genomes, and the original known host lineage for each prophage were both retrieved from the Virus-Host DB (https://www.genome.jp/virushostdb/). In addition, the host of each prophage were further evaluated by using a web based tool called HostPhinder (https://cge.cbs.dtu.dk/services/HostPhinder/) [45].

### Comparative phage genome analysis

All 142 phage genome sequences were pooled and saved as a multi-fasta file and aligned to one another using MUMmer v4.0.0.beta2. Genome comparisons were carried out to produce delta files using the following breaklen parameters: maxgap = 200; mincluster = 90; minmatch = 60. Results were generated as coordinate files using “shwon-coords” and visualized via Circos [46]. To illustrate the nucleotide similiarities among all the analyzed phage genomes, the visualization tool Circos [46] that display connecting lines between prophages was used. Whole genome alignment and calculation of percentage of ANI identity were carried out with Kalign [30]. Evolutionary analyses for tree construction were conducted in MEGA X [47]. Briefly, the evolutionary history was inferred using the Neighbor-Joining method [48]. The bootstrap consensus tree inferred from 500 replicates [49] was taken to represent the evolutionary history of the taxa analyzed. Branches corresponding to partitions reproduced in less than 50% of bootstrap replicates were collapsed. The percentage of replicate trees in which the associated taxa clustered together in the bootstrap test (500 replicates) was shown next to the branches. The evolutionary distances were computed using the Maximum Composite Likelihood method [50] and are presented as the number of base substitutions per site. There were a total of 431,295 positions in the final dataset. Prophage genomes (*n* = 142 phage) were concatenated into a single sequence with a total length of 7,260,982 bp, which when plotted against itself with a sliding window of 10 bp and visualized by Gepard 1.40 version [29], revealed an overall pattern of similarity or dissimilarity of all the genomes. The graphics displayed pairwise similarity between genomes which was then used for the preliminary assignment of clusters. Among all the analyzed prophage genomes, if two sequences shared high similarity, a diagonal would show at that location on the plot (the center diagonal line demonstrated the 100% similarities where a sequences was compared to itself).

### Genome clustering

Three criteria were used to cluster the phage genomes. First, the genomes were grouped based on nucleotide identity among members. Second, dot plot was used to analyze sequences based on similarity leading to graphically demonstrable clustering of sequences. Third, translated nucleotide sequences were used to cluster phages based on translated amino acids sequences. Phage genomes that did not meet these criteria were identified as ‘singletons’.

### *Salmonella* phage database creation and genome map viewing via Phamerator

In order to produce the first, web-based inventory of *Salmonella* prophages that could be used for comparative analysis with prophage genomes from other bacteria, we created the SpDB in the Pharmerator platform. For this purpose, *Salmonella* phage database, a web-based application PhamDB was used for building the *Salmonella* Phamerator phage database consisting of 142 phage sequences. Briefly, after installing Docker Toolbox, Kitematic was launched to finish the initial setup and loading. An existing ‘PhamDB’ database in the Phamerator platform was downloaded and used as a template. By running the PhamDB program as a web interface on a local network, a new database was created in toolbar using GenBank Files as inputs. All the 142 phage NCBI files were summarized in Document S2. The generated database was a sql file which was used as an input file and uploaded into Phamerator website (https://phamerator.org, created and maintained by Dr. Steven Cresawn of James Madison University). Based on the assigned clusters, genome maps can be visualized for direct comparisons. As displayed in the Phamerator map, long regions of violet shading indicate long conserved regions between phage genomes. Within a cluster, the same color block represents the ORF with higher similarities. Regions of high similarity and same-coloured ORF blocks shown on the map indicated a prevalent synteny. Areas with little or no sequence similarity between genome sequences are shown as either white blocks or a break in a syntenic block.

## Supplementary information

**Additional file 1: Figure S1.** Phylogenetic tree of 142 *Salmonella* prophages based on genome alignment and nucleotide identity using Kalign. The relationships among the *Salmonella* isolates based on genome alignments and % nucleotide identity were inferred using the Neighbor-Joining method and conducted in MEGA X.

**Additional file 2: Table S1.** Detailed profiles of 142 prophages present in *Salmonella enterica.*

**Additional file 3: Table S2.** Nucleotide similarities between pairs of *Salmonella* prophage genomes (*n* = 142).

**Additional file 4: Table S3.** Nucleotide identify matrix for 17 clusters of Cluster A-Q.

**Additional file 5: Table S4.** All the assigned Pham numbers and its color code.

**Additional file 6: Table S5.** The distribution of conserved Phamily members among clusters of *Salmonella* bacteriophages.

**Additional file 7: Document S1.** Multi-fasta files for the 142 *Salmonella* prophage sequences.

**Additional file 8: Document S2.** NCBI files of 142 prophages used for Phamerator database creation.

**Additional file 9: Document S3.** Scalable Vector Graphics (SVG) figure demonstration files for the 17 assigned clusters of 142 prophages.

## Data Availability

The datasets supporting the conclusions of this article are included within the article and its additional files.

## Abbreviations

ORFs: Open Reading Frames
PHASTER: PHAge Search Tool Enhanced Release
CRISPRs: clustered regularly interspaced short palindromic repeats
ICTV: International Committee on Taxonomy of Viruses
Gepard: Genome Pair Rapid Dotter
dsDNA: Double stranded DNA
ssDNA: Single stranded DNA
