# Supplementary figures and images for: Comparative genomic analysis of 142 bacteriophages infecting Salmonella enterica subsp. enterica
Source: BMC Genomics. 2020 May 26;21:374. doi: 10.1186/s12864-020-6765-z (PMC7251866; doi:10.1186/s12864-020-6765-z)

## Slide 1
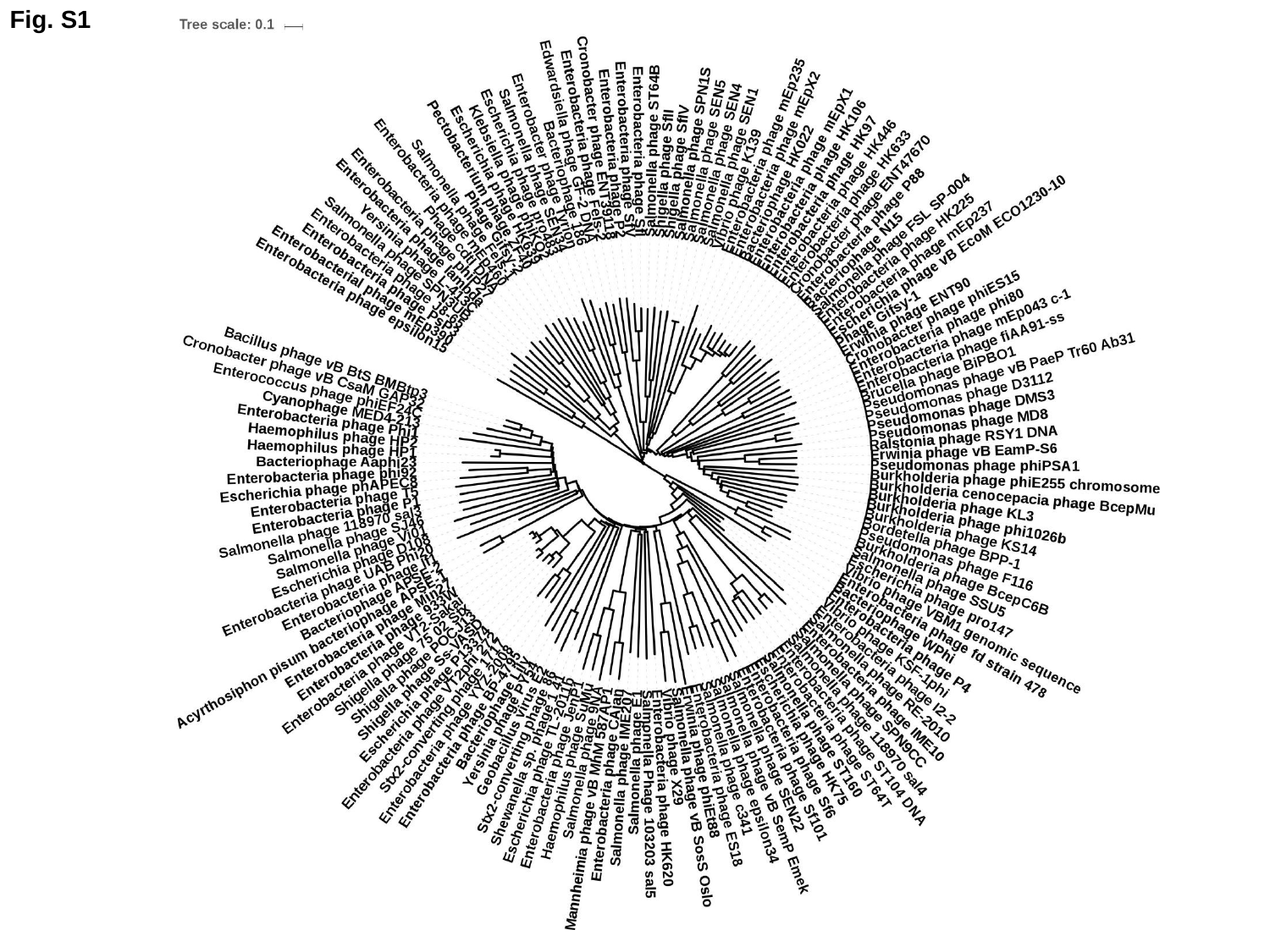

Fig. S1

Supplement: Supplementary file 1 — Additional file 1: Figure S1. Phylogenetic tree of 142 Salmonella prophages based on genome alignment and nucleotide identity using Kalign. The relationships among the Salmonella isolates based on genome alignments and % nucleotide identity were inferred using the Neighbor-Joining method and conducted in MEGA X. [file 12864_2020_6765_MOESM1_ESM.pptx]
